# Immigrants’ Access to Health Insurance: No Equality without Awareness

**DOI:** 10.3390/ijerph110707144

**Published:** 2014-07-14

**Authors:** Dagmar Dzúrová, Petr Winkler, Dušan Drbohlav

**Affiliations:** 1 Department of Social Geography and Regional Development, Faculty of Science, Charles University in Prague, Albertov 6, Prague 128 43, Czech Republic; E-Mails: dzurova@natur.cuni.cz (D.D.); drbohlav@natur.cuni.cz (D.D.); 2 Prague Psychiatric Centre, Department of Social Psychiatry, National Institute of Mental Health, Ustavni 91, Prague 181 03, Czech Republic

**Keywords:** migration, access to health care, health insurance, health policy evaluation, cross-sectional survey, health care awareness

## Abstract

The Czech government has identified commercial health insurance as one of the major problems for migrants’ access to health care. Non-EU immigrants are eligible for public health insurance only if they have employee status or permanent residency. The present study examined migrants’ access to the public health insurance system in Czechia. A cross-sectional survey of 909 immigrants from Ukraine and Vietnam was conducted in March and May 2013, and binary logistic regression was applied in data analysis. Among immigrants entitled to Czech public health insurance due to permanent residency/asylum, 30% were out of the public health insurance system, and of those entitled by their employment status, 50% were out of the system. Migrants with a poor knowledge of the Czech language are more likely to remain excluded from the system of public health insurance. Instead, they either remain in the commercial health insurance system or they simultaneously pay for both commercial and public health insurance, which is highly disadvantageous. Since there are no reasonable grounds to stay outside the public health insurance, it is concluded that it is lack of awareness that keeps eligible immigrants from entering the system. It is suggested that no equal access to health care exists without sufficient awareness about health care system.

## 1. Introduction

Migration is perceived as a great challenge for public health in the globalizing world [1]. The phenomenon of migrants’ limited access to health care has been known for a long time [2] and institutions such as the WHO and European Union aim to address it [3,4,5]. The amount of evidence that migrants face inequalities in health is, however, increasing [6,7] and evidence suggests that inequalities cannot be explained merely by immigrants’ lower social position [8,9]. This is particularly worrying for two reasons. First, it has been shown that the “healthy migrant effect” tends to disappear in the long run [7], and second, the field of health equity has been, at least in Europe, dominated by an approach targeting socioeconomic factors while marginalizing ethnicity, migration and other factors [10].

Since the Velvet Revolution, a total transformation of the whole society—from a socialist/communist regime to a democratic, parliamentary system based on a free market economy—has started in Czechia. This transformation process, along with ongoing globalization and the shift towards a post-modern society has brought significant inflows of immigrants [11]. Over time, the number of immigrants grew and reached slightly more than 400,000 legally registered immigrants in 2008. Since then this figure has stayed more or less constant and stood at 434,153 foreigners as of 31 December 2011 (out of this number 151,276 immigrants came from the EU while 282,877 were non-EU nationals [12]). The most numerous immigrant group is Ukrainians (118,058 as of the very end of 2011). The intensive migratory inflow and high numbers of immigrants in the country (stocks) attracted attention in research circles. Thus, various aspects of immigrants’ integration in Czechia have been studied—see e.g., Vavrečková [13], Brabcová [14], Jelínková [15], Vacková [16], Pikhart [17], Křečková-Tůmová [18], Nesvadbová [19]. It seems, however, that relatively little attention has been paid to issues related to immigrants’ health, in general, and immigrants’ health insurance, in particular [20,21].

In Czechia, there are three stages of immigration process applicable to third-country nationals (non-EU immigrants) (this paper is not concerned with citizens of the EU states and Norway, Switzerland, Iceland and Liechtenstein and their dependants; first, they did not enter our survey and moreover, their legal conditions are more or less comparable with those faced by the Czech majority population) which are representative for vast majority of non-EU citizens on the territory of Czechia [12]. Three types of immigrants’ group may be identified according these stages: (1) non-EU immigrants who stay on the basis of a long-term visa for over 90 days. Maximum validity of this visa is 6 months, it cannot be prolonged, but it might be followed by a long-term residence permit; (2) non-EU immigrants who obtained a long-term residence permit which either followed a long-term visa or the application for this permit at an embassy or consulate of the Czech Republic abroad. In the latter case, this permit is given without a previous stay on a long-term visa. Long-term residence permits may be granted for a period longer than 1 year and its validity may be prolonged [12]. The aforementioned two categories are often economically based and frequently correspond to either employed or self-employed (small entrepreneur) status. (3) finally, there are non-EU immigrants who obtained permanent residence permits. Permanent residence permits often accompany family-related migration (family reunion or family creation), or are a result of aconsiderably long and complex process of integration into Czech society. As already mentioned, non-EU immigrants can be granted this status under special circumstances. The three aforementioned groups does not include non-EU migrants who stay in Czechia for a short time—be it with or without a short-term visa which is valid for up to 90 days—nor do they include those non-EU migrants who hold asylum status or subsidiary or temporary protections—asylum status is awarded somewhat exceptionally in Czechia. Only 1603 asylum seekers were admitted with asylum status in 10 years between 2001 and 2010, and there were only 20 Vietnamese and 112 Ukrainians among them [22]. Totally, there were only 174 Vietnamese and 171 Ukrainians holding asylum status in Czechia in 2011 [22]. The current process of obtaining visa/permits depends on both the aforementioned migratory type and position on the labor market, it is accompanied by a number of prerequisites which have to be met such as providing biometric data, proof of qualification, accommodation, financial sources and health insurance.

Public health insurance is crucial for access to health care in Czechia. It offers almost universal coverage, it is a major source of funding for health care [23], and it is compulsory for those specified by law including all legal migrants with permanent residency status, all long-term (more than 90 days) migrants from the EU, and all migrants with employee status [20]. Non-EU nationals (who are of concern in the present study) can apply for permanent residence: (i) “Generally, after 5 years of continuous residence in Czechia; (ii) After 4 years of continuous residence provided that the foreigner has been granted a temporary residence permit after the termination of proceedings for the granting of international protection; (iii) Regardless of the length of the previous stay provided that the foreigner applies for permanent residence for humanitarian reasons or other reasons deserving special attention, in the interest of Czechia or provided that the applicant is a minor or an adult dependant of a foreigner with permanent residence on the territory for the purpose of joint cohabitation of the family” [12]). All other migrants, however, are not eligible for public health insurance and they are therefore forced to enter a disadvantageous system of commercial health insurance [20] or they remain without any insurance, which against the law. Commercial health insurance has been heavily criticized on the basis of its discriminatory nature towards those who come from countries outside the EU [21].

In the context of the above, the present study among non-EU immigrants was conducted and data gathered in March and May 2013. The main goal was to examine immigrants’ access to public health insurance in Czechia. The subsequent goal was to determine what are the barriers that prevent immigrants from utilizing health care benefits even if they are de iure entitled for them. This endeavor should also be considered as an evaluation of objectives declared in the Czech Government Resolution from 2009. This resolution stated that immigrants’ equal access to public health insurance as well as the issue of migrants’ low level of awareness were major concerns to be solved as soon as possible.

## 2. Methods

A questionnaire survey of two immigrant groups in Czechia—Ukrainians and Vietnamese—was carried out between March and May 2013 and a total of 912 respondents were successfully contacted. Ukrainians and Vietnamese were chosen as they are the most populous migrant groups in Czechia. A quota sampling method was used with quota characteristics [24] represented by sex, age and region of migrant’s residence. The sample included immigrants who have already been in Czechia for more than 1 year. Those who have moved at least once inside Czechia were intentionally overrepresented (the main research task was to research the internal mobility of immigrants). The data were collected through the interviewer network of The Center of Independent Public Opinion Research (under the umbrella of the Institute of Sociology of the Academy of Sciences of the Czech Republic). The aim of the interviewers was to find appropriate respondents (they were provided with information about locations where immigrants may be concentrated), to distribute questionnaires and then to collect them. The respondents filled in the questionnaire (via self-administered mode) in their mother tongue and it took on average about 45 min.

Multivariate analysis was utilized in order to analyze a comprehensive set of variables. This analysis allowed to identify the relationship between variables and to suggest, with reasonable degree of validity, why immigrants entitled for PHI have or have not PHI. The analysis was undertaken using SPSS Statistics version 17.0 (SPSS Inc., Chicago, IL, USA). Binary logistic regression was applied in order to examine the independent effects of the sociodemographic and migrant variables on coverage of public health insurance (PHI) for the group of immigrants entitled to PHI (dichotomized in two categories: 1—he/she has not got PHI, 0—he/she has got PHI). Seven independent variables were included in the model. Fully adjusted odds ratios (OR) and 95% confidence intervals [25] were computed by using the beta coefficients and standard errors.

Age was categorized into three age groups: 18–30 years, 31–40 years and 41–62 years. Education was categorized as basic (or less), complete secondary education, and complete university education. In relation to marital status, people were also classified into three categories: (i) married/cohabitated and partner lives in Czechia [3]; (ii) married/cohabitated and partner does not live in Czechia and (iii) other possibilities (single, divorced, or widowed).

Knowledge of the Czech language was tested via the following statement: “I am able to communicate in Czech”. Answering options were: “I definitely agree”, “I agree”, “I do not agree”, and “I strongly disagree”. Those reporting “I definitely agree” were considered as “Good”, “I agree” as “Average” and “I do not agree, and strongly disagree” as “Bad”.

The level of wages was tested by the following question: “What is currently the average net monthly income of all household members in the Czech Republic?” Respondents were allowed to choose one of eight options (No income; Less than 10,000 CZK; 10,000 to 14,999 CZK; 15,000 to 19,999 CZK; 20,000 to 29,999 CZK; 30,000 to 39,999 CZK; 40,000 to 69,999 CZK and more than 70,000 CZK) and responses were categorized into three categories (as low, with less than 5,000 CZK, average with 5001 to 12,501 CZK, and high with 12,501 CZK or more).

## 3. Results

Table 1 shows that half of the non-EU immigrants, represented in this study by those from Ukraine and Vietnam, were insured under the public health insurance system (50.8%). The rest of the sample had commercial health insurance (37.2%) or were not insured at all (12%).

**Table 1:** Migrants by health insurance, type of residence and employment. PHI—public health insurance, CHI—commercial health insurance.

Migrants’ Characteristics	Health Insurance			Total
	PHI	CHI	Without	
Long-term stay—*n* (%)	180 (38.4)	214 (45.6)	75 (16.0)	469 (100)
Permanent stay or asylum—*n* (%)	269 (69.9)	107 (27.8)	9 (2.3)	385 (100)
Other type of residence—*n* (%)	11 (21.2)	16 (30.8)	25 (48.1)	52 (100)
Employee—*n* (%)	167 (50.0)	118 (35.3)	49 (14.7)	334 (100)
Enterpreneur—*n* (%)	139 (49.3)	122 (43.3)	21 (7.4)	282 (100)
Other type of employment—*n* (%)	154 (53.5)	96 (33.3)	38 (13.2)	288 (100)
Total—*n* (%)	460 (50.8)	337 (37.2)	109 (12.0)	906 (100)

Since there is a right and, in fact, a legal obligation to enter the PHI system for individuals who have permanent residence status and/or are officially employed, there are some evident discrepancies revealed by this survey. 30% of immigrants with permanent residence status and 50% of immigrants with employee status have not yet entered the PHI system. Both requirements for entering the public health insurance service (permanent residence status and employee status) were met simultaneously by 10% of immigrants in the sample (*n* = 98). While 73.6% (72) were insured via the PHI, the rest of the sample (26.4%) was not.

Table 2 shows results of the multivariable analysis measuring the association between migrant characteristics and the presence of health insurance for two sub-groups of migrants. The first logistic model examines the independent effects of the sociodemographic and migratory variables on registration for health insurance within the group of migrants with permanent residence status. The results tell us that migrants with a poor knowledge of the Czech language were significantly more likely to be without/uncovered by PHI. In particular, migrants with a poor knowledge of the Czech language were more than two times more likely to be without PHI than migrants with a good knowledge of Czech (OR = 2.23, 95% CI 1.03, 4.86). When examining differences by marital status, married migrants with a partner outside Czechia were almost three times more likely not to have PHI cover compared to single migrants (OR = 2.71, 95% CI 1.21, 6.06). Other independent variables were not confirmed as significant.

The second logistic model (Table 2) shows the effects of independent variables on registration for health insurance within a group of migrants with employee status. The results provide almost the same conclusions as those revealed by the first model. Uninsured immigrants were those with a poor knowledge of the Czech language and the strength of this relationship is even more marked. Migrants with a poor knowledge of Czech were almost three times likely to be uncovered by PHI than migrants with a good level of Czech (OR = 2.74, 95% CI 1.12, 6.75). Differences in PHI coverage between migrants of different marital status were not confirmed here. Other independent variables did not enter the model either.

**Table 2:** Adjusted odds ratio and 95% CI for coverage with public health insurance for the group of immigrants entitled to PHI.

Dependent Variable: 1 = Has not Got PHI; 0 = Has Got PHI	Model 1 Migrants—Permanent Stay or Asylum *n* = 385			Model 2 Migrants—Employee *n* = 334		
7 Independent Variables	Adj. OR	95% CI		Adj. OR	95% CI	
		Lower	Upper		Lower	Upper
Females—REF	1			1		
Males	1.193	0.748	1.902	0.885	0.539	1.454
Vietnamese—REF	1			1		
Ukrainians	0.959	0.573	1.606	0.533	0.271	1.049
Czech language—good REF	1			1		
Czech language—average	1.495	0.902	2.477	**1.846**	**1.126**	**3.026**
Czech language—bad	**2.233**	**1.027**	**4.855**	**2.744**	**1.115**	**6.751**
Wages—low REF	1			1		
Wages—average	1.245	0.717	2.164	0.830	0.411	1.675
Wages—high	1.566	0.755	3.248	0.541	0.240	1.219
Marital status—other REF	1			1		
Married—partner not in CZ	**2.712**	**1.213**	**6.061**	1.131	0.567	2.256
Married—partner in CZ	1.096	0.603	1.994	0.416	0.230	0.751
Education—university REF	1			1		
Education—elmentary	1.480	0.593	3.689	2.183	0.915	5.205
Education—secondary	1.459	0.579	3.678	1.612	0.676	3.845
Age < 31	0.829	0.439	1.565	1.146	0.607	2.164
Age 31–40	0.850	0.471	1.534	0.892	0.499	1.596
Age > 40 REF	1			1		

Note: Bold when *p* < 0.05.

## 4. Discussion and Conclusions

This study is a pioneering attempt to utilize statistical analysis of a relatively large group of respondents in order to determine factors and decisive moments behind immigrants’ health behavior. This was demonstrated on a large sample of non-EU immigrants in Czechia and their [26] involvement in the public health insurance system. Significant disparities in health insurance and immigrants’ seemingly irrational health behavior were revealed among non-EU migrants. The immigrants’ limited access to public health insurance, even when they are entitled to it, is alarming and contrary to what is economically and also legally far more reasonable in terms of health protection. This also shows that the need for equal opportunities, particularly in access to health care, and integration into Czech society as declared in the Conception of Integration of Foreigners Revised [27] have not yet been met. From the political perspective, the health insurance gaps for immigrants remain of great concern.

The findings of the present study may face several limitations. First, some of the respondents may not have been clear what kind of health insurance system they belong to. This, however, would be highly improbable for such a great number of respondents, *i.e.*, 30% of those with a permanent residence status and 50% of those with employee status. Second, some of the respondents who have been found to still be in the system of commercial insurance or uninsured despite the fact they were entitled to public health insurance on the basis of their employment status, might actually have reported employment status, although they were working irregularly. This might have been true for many of the respondents, but again, it is highly improbable to be so for all of the 50% of those with employment status. Third, in Czechia, when immigrants ask for permission to stay in the country, they have to prove they are insured for the whole length of intended stay. This forces them to buy commercial insurance for the whole length of intended stay, most often for one year. Immigrants are not allowed to sign off the commercial contract, no matter if they, in the meantime, become employees or get permanent residency and thus enter the system of public health insurance. It might be that there is a considerable group of migrants who are forced to pay for both commercial and public health insurance, which is clearly discriminatory and widely criticized. Again, in the context of the present survey, it is not probable that this could explain the identified discrepancies, as the sample consisted of those immigrants who were in country for more than one year.

This study raises a question of what makes equal health care opportunities. The present analysis focused on migrants with theoretically equal access to health care—all of them were entitled to public health insurance, which offers nearly universal coverage. Despite this, a considerable minority have remained registered in the commercial health insurance system. Besides being married to a partner living outside Czechia, only a poor knowledge of the Czech language has been clearly identified as a risk factor for not entering the public health insurance system. Such findings suggest that it is probably low awareness of opportunities and insufficient orientation (and insufficient and poorly targeted information) in the society that restrains migrants from accessing equal opportunities. This could have a fundamental implication for health promotion. Immigrants are, of course, not to be blamed for their lack of awareness, rather individual countries shall introduce programs to empower immigrants and increase their health literacy. Countries shall understand that health care systems require people to understand and use health information and that this might be challenging even for natives [25,28] and so much the more for immigrants who often face language, cultural and economic difficulties [29]. It is thus of great importance to include health awareness and health literacy activities into related national action plans and programs as it has been already done for example in Switzerland [30]. Furthermore, it is important to appropriately communicate relevant health care information at each stage of the migration process, and this should be an integral part of migrant health policies as defined and suggested, for instance, by Zimmerman, *et al.* [31] or Rechel *et al.* [32].

In recent years, health promotion has generally been strongly focused on reducing inequalities, integrating health into public policies and making people to change their lifestyles [5,33]. This study suggests that migrants do not benefit from positive steps in health promotion (involvement in the public health insurance system) and even disadvantage themselves purely because of lack of awareness. Theoretical equal access to health care is thus not enough in real world, unless it is accompanied by sufficient awareness of the part of migrants about the health care system of the host country. Further research should investigate possible efficient ways to raise migrants’ awareness of their equal opportunities and to empower them to utilize these.
